# The Effect of Hydrodynamic Cavitation on the Structural and Functional Properties of Soy Protein Isolate–Lignan/Stilbene Polyphenol Conjugates

**DOI:** 10.3390/foods13223609

**Published:** 2024-11-12

**Authors:** Ning Hua, Xian’e Ren, Feng Yang, Yongchun Huang, Fengyan Wei, Lihui Yang

**Affiliations:** 1 Guangxi Key Laboratory of Green Processing of Sugar Resources, Key Laboratory for Processing of Sugar Resources of Guangxi Higher Education Institutes, School of Biological and Chemical Engineering, Guangxi University of Science and Technology, Liuzhou 545006, China; 13057171823@163.com (N.H.); yfyangfeng@hotmail.com (F.Y.); huangyc@yeah.net (Y.H.); weifengyan2022@163.com (F.W.); yanglihui0121@126.com (L.Y.); 2 Guangxi Liuzhou Luosifen Research Center of Engineering Technology, Liuzhou 545006, China

**Keywords:** hydrodynamic cavitation, SPI, polyphenols, structure, emulsifying properties, antioxidant capacity

## Abstract

In this study, hydrodynamic cavitation technology was utilized to prepare conjugates of soy protein isolate (SPI) with polyphenols, including resveratrol (RA) and polydatin (PD) from the stilbene category, as well as arctiin (AC) and magnolol (MN) from the lignan category. To investigate the effects of hydrodynamic cavitation treatment on the interactions between SPI and these polyphenols, the polyphenol binding capacity with SPI was measured and the changes in the exposed sulfhydryl and free amino contents were analyzed. Various methods, including ultraviolet–visible spectroscopy, fluorescence spectroscopy, Fourier transform infrared spectroscopy, and circular dichroism spectroscopy, were also used to characterize the structural properties of the SPI–polyphenol conjugates. The results showed that compared to untreated SPI, SPI treated with hydrodynamic cavitation exposed more active groups, facilitating a greater binding capacity with the polyphenols. After the hydrodynamic cavitation treatment, the ultraviolet–visible absorption of the SPI–polyphenol conjugates increased while the fluorescence intensity decreased. Additionally, the content of exposed sulfhydryl and free amino groups declined, and changes in the secondary structure were observed, characterized by an increase in the α-helix and random coil content accompanied by a decrease in the β-sheet and β-turn content. Furthermore, the SPI–polyphenol conjugates treated with hydrodynamic cavitation demonstrated improved emulsifying characteristics and antioxidant activity. As a result, hydrodynamic cavitation could be identified as an innovative technique for the preparation of protein–phenolic conjugates.

## 1. Introduction

Due to population growth, the demand for protein is gradually increasing. By 2050, the global population will reach 9.8 billion, leading to a projected increase in protein demand of approximately 70% [1,2]. The extensive production of animal protein can result in ecological and environmental issues, such as the overconsumption of water and land resources and contributing to the greenhouse effect. Plant-based proteins offer advantages such as low fat content, rich nutritional value, cost-effectiveness, and environmental sustainability while possessing functional characteristics similar to those of animal proteins. Utilizing plant proteins as substitutes for certain animal proteins has become a trend to address these challenges. Soybeans are a significant protein source, comprising around 40% protein [3]. Soy protein isolate (SPI) is derived from soybeans and contains more than 90% protein, featuring a comprehensive amino acid profile and possessing processing and functional properties, including solubility, emulsification, and water retention, which contribute to its extensive use in the food industry [4]. However, SPI also has some drawbacks, such as a weak antioxidant capacity and sensitivity to environmental conditions (pH, metal ions, etc.), which complicate processing and storage conditions, hindering its further development [5]. As a result, an increasing number of researchers are modifying SPI to improve its functional properties, enabling a broader variety of applications in food processing.

In recent years, the interaction between polyphenols and proteins to improve protein structure and functional properties has become a focal point in the research of functional food proteins. Polyphenolic compounds exhibit various bioactive functions, including antioxidant, antibacterial, anticancer, and immune-enhancing activities. Studies have shown that polyphenols can bind to proteins through non-covalent interactions, such as hydrogen bonds, ionic bonds, van der Waals forces, and hydrophobic interactions. Additionally, polyphenols can covalently bond with proteins via C-N or C-S bonds under alkaline conditions, enzymatic catalysis, or free radical induction. The interaction between proteins and polyphenols not only modulates the functional properties of the proteins, but also imparts certain bioactivities, presenting promising applications. Research by Dai et al. [6] found that catechins could non-covalently bind to soy protein via hydrophobic interactions and hydrogen bonding. This binding leads to a more loose and disordered protein structure, with a transformation of the secondary structures from α-helices and β-sheets to β-turns and random coils. Findings by Tong et al. [7] indicated that epigallocatechin gallate could bind to soy protein through hydrogen bonding and hydrophobic interactions, enhancing its stability, viscosity, and antioxidant activity. Wang et al. [8] utilized laccase to catalyze the covalent coupling of gallic acid to soy protein, which altered the protein’s secondary structure and led to a notable enhancement in its antioxidant activity. These studies indicated that polyphenols with different structural characteristics could lead to varying conformational changes in proteins, thus affecting their functional properties.

**Figure 1:**
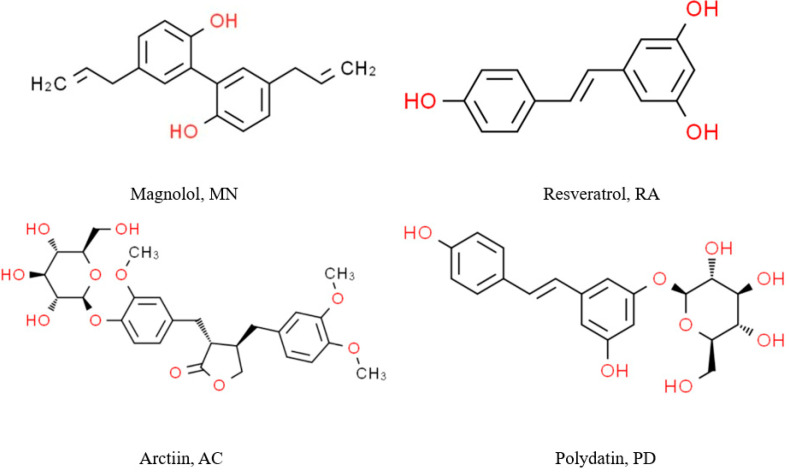
Structures of stilbene polyphenols and lignan polyphenols.

Polyphenolic compounds can be categorized into four classes based on their structures: phenolic acids, flavonoids, lignans, and stilbenes. Differences in molecular weight, hydroxylation, methylation, hydrogenation, and glycosylation among the various phenolic compounds significantly influence how the polyphenols interact with proteins. Currently, there is extensive research on the binding of flavonoids and phenolic acids with proteins, whereas reports on lignans and stilbenes are relatively scarce. Therefore, we selected two types of lignan polyphenols (magnolol (MN) and arctiin (AC)) and two types of stilbene polyphenols (resveratrol (RA) and polydatin (PD)) (with the polyphenol structures shown in Figure 1) to study the differences in the interaction patterns between polyphenols with distinct molecular structures and SPI, as well as their effects on protein structure and functional properties. In addition, we introduced hydrodynamic cavitation technology to treat the SPI–polyphenol conjugates and studied the effects of hydrodynamic cavitation the on SPI–polyphenol conjugates.

Hydrodynamic cavitation, as a novel physical processing technology in food science, generates cavitation effects similar to those produced by ultrasound. During the hydrodynamic cavitation process, significant pressure fluctuations can occur as the fluid passes through orifices or Venturi tubes, or as a result of rotor or impeller action, leading to cavitation bubbles. The collapse of these cavitation bubbles generates effects such as shear forces, shock waves, extreme temperatures and pressures, and free radicals. Compared to micro-fluidization technology, the orifices used in hydrodynamic cavitation are primarily designed to generate cavitation bubbles, while the orifices in micro-fluidization are intended to produce high-velocity fluids. This setup, combined with the interaction chamber, facilitates the formation of small and uniform particles [9]. Micro-fluidization is a high-pressure homogenization technology with a lower energy efficiency, making it suitable for precise applications [10]. Moreover, hydrodynamic cavitation offers advantages over ultrasonic cavitation, including a higher energy efficiency, ease of scaling up, and more significant industrial application potential [11]. There have been many research reports on the application of hydrodynamic cavitation in protein modification. For instance, Gregersen et al. [12] discovered that applying hydrodynamic cavitation to modify whey protein concentrate resulted in the depolymerization of large aggregates, a significant decrease in viscosity and particle size, and an enhanced storage stability. Additionally, research by Asaithambi et al. [13] demonstrated that hydrodynamic cavitation could unfold the structural configuration of egg white protein hydrolysates, improving the degree of protein hydrolysis and surface hydrophobicity, ultimately enhancing the nutritional and functional properties of the egg white protein hydrolysates. The preliminary research findings from our team indicated that the treatment of SPI with hydrodynamic cavitation (H-SPI) resulted in a reduction in particle size, an increase in surface hydrophobicity, the cleavage of disulfide bonds, the disruption of higher-order structures, and a loosening of the previously compact structure. Perhaps the disruption and unfolding of this structure can enhance the binding of SPI with polyphenols, while hydrodynamic cavitation can generate free radicals, which have been reported to promote the covalent bonding of polyphenols with SPI. However, there have been limited studies addressing the use of hydrodynamic cavitation technology to enhance the interactions that occur between proteins and polyphenols. Building upon this, the present study further utilizes the shear and radical effects of hydrodynamic cavitation to induce the depolymerization of SPI, exposing more active sites for binding with polyphenols of varying structures. Under the continuous action of the generated free radicals, we aim to facilitate the covalent bonding of polyphenols with SPI to form SPI–polyphenol conjugates. The characterization of the structural and functional properties of these conjugates will provide new technological avenues for the development of functional soy proteins.

## 2. Materials and Methods

### 2.1. Materials

SPI (91.1% of protein) was purchased from Shandong Yuwang Eco-Food Industry Co., Ltd. (Dezhou, China). MN (98%), RA (99%), and PD (98%) were acquired from Aladdin (Shanghai, China). AC (98%) was obtained from Xi’an Huiling Bio-Tech Co., Ltd. (Xi’an, China). 2,2-diphenyl-1-picrylhydrazyl (DPPH, 96%) and 2,2′-azino-bis (3-ethylbenzothiazoline-6-sulfonic acid) (ABTS, 98%) were purchased from Macklin (Shanghai, China). All other reagents used in the study were of analytical quality.

### 2.2. Preparation of SPI–Lignan/Stilbene Polyphenols

SPI powder (2%, *w*/*v*) was dissolved in deionized water and magnetically stirred for 2 h at 25 °C to allow for sufficient dissolution. The four polyphenols (MN, AC, RA, and PD) were then separately dissolved in anhydrous ethanol (1%, *w*/*v*) and fully mixed under dark conditions. Subsequently, the polyphenol solutions were added to the SPI dispersion (2%, *w*/*v*), and the pH was adjusted to 7.0. Deionized water was then used to dilute the mixture to achieve final concentrations of 1% and 0.1% (*w*/*v*) for SPI and polyphenols, respectively.

**Figure 2:**
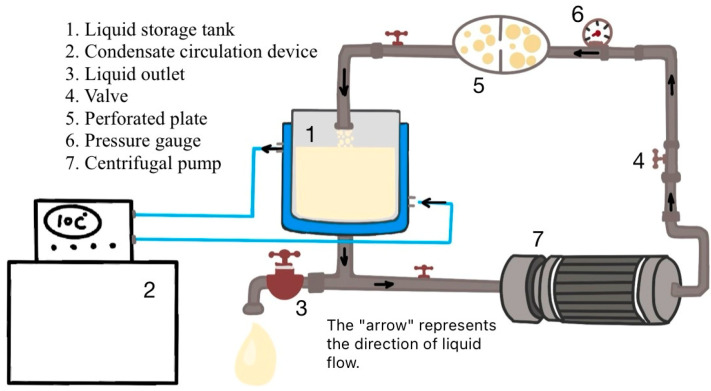
Schematic diagram of hydrodynamic cavitation device structure.

The hydrodynamic cavitation device consists of a centrifugal pump (power 550 W), a storage tank, a condensate circulation device, a perforated plate (with a hole diameter of 3 mm), a pressure gauge, valves, and piping (18 mm). A schematic diagram of the device is shown in Figure 2. When the fluid passes through the perforated plate, its flow velocity suddenly increases, and the pressure decreases. When the pressure drops below the saturated vapor pressure of the fluid, the fluid vaporizes, and the gas dissolved in the fluid is released, generating a large number of cavitation bubbles. As the fluid flows through the pressure recovery zone, the collapse of these cavitation bubbles produces effects such as shear force, shock waves, extremely high temperatures and pressures, and free radicals.

The mixture was placed in the storage tank of the hydrodynamic cavitation device with the power turned on and the condensate circulating, ensuring that the temperature of the mixture did not exceed 35 °C during the treatment, which lasted for 30 min. At the same time, a control group was established where the mixture was not subjected to the hydrodynamic cavitation treatment and was stirred magnetically for 30 min instead. After treatment, the mixture was permitted to sit for 12 h, followed by centrifugation at 8000 r/min for 20 min. After collecting the supernatant, it was dialyzed against deionized water through a dialysis bag with a molecular weight cut-off of 3500 to remove ethanol and unbound polyphenols from the mixture, resulting in a liquid sample of the SPI–polyphenol conjugate, which was then stored in the refrigerator for later use [14]. The samples treated with hydrodynamic cavitation were labelled as the H group, specifically H-SPI, H-SPI-MN, H-SPI-RA, H-SPI-AC, and H-SPI-PD. The samples that were not subjected to the hydrodynamic cavitation treatment were labelled as the UH group, namely UH-SPI, UH-SPI-MN, UH-SPI-RA, UH-SPI-AC, and UH-SPI-PD.

### 2.3. Binding Capacity of Polyphenols to Proteins

The binding capacity of polyphenols to proteins was determined using a modified Folin–Ciocalteu method [15]. A sample (1 mL, with an SPI concentration of 2 mg/mL) was reacted with Folin–Ciocalteu reagent (0.5 mL, 0.2 mol/L) for 5 min in the dark. After this, Na_2_CO_3_ (3 mL, 5%, *w*/*w*) was added, and the reaction was continued in the dark for 30 min. The absorbance was then measured at a wavelength of 760 nm. A standard curve for the polyphenols was established, and the amount of polyphenols in the SPI–polyphenol conjugates was calculated based on this standard curve (MN: y = 7.0411x + 0.1291, R^2^ = 0.9993; RA: y = 23.564x + 0.1503, R^2^ = 0.9954; AC: y = 4.3441x + 0.042, R^2^ = 0.9864; PD: y = 12.872x + 0.0332, R^2^ = 0.997). The calculation was conducted using Formula (1):

(1) $$\mathrm{Binding}\;\mathrm{Capacity}\;\mathrm{of}\;\mathrm{Polyphenols}\;\mathrm{to}\;\mathrm{SPI}\;(\mathrm{mg}/g)=\frac{M_{1}}{M_{2}},$$

where *M*_1_ represents the content of polyphenols in the polyphenol–protein conjugates (mg), while *M*_2_ represents the content of SPI in the sample mixture (g).

### 2.4. Determination of Free Amino and Exposed Sulfhydryl Content

Following the methodology of Liu et al. [16], the content of free amino groups was measured using the Ortho-Phthalaldehyde (OPA) method. Initially, an OPA reagent was prepared by dissolving 4 mL of 40 mg/mL OPA solution in methanol, then adding 100 mL of 0.1 mol/L borate buffer, 400 μL of mercaptoethanol, and 10 mL of 200 mg/mL sodium dodecyl sulfate solution (SDS), and finally adjusting the volume to 200 mL. The sample was determined by reacting 400 μL of the sample with 3 mL of the OPA reagent in a water bath at 35 °C for 3 min. The absorbance was measured at a wavelength of 340 nm. A standard curve for L-leucine was generated to calculate the content of free amino groups.

Following the method described by Liu et al. [17], Tris–glycine buffer and Ellman’s reagent were prepared. A sample (1 mL, 10 mg/mL) was uniformly mixed with the Tris–glycine buffer (4 mL), to which the Ellman’s reagent (50 μL) was added. The mixture was allowed to stand at room temperature for 20 min, and the absorbance was measured at a wavelength of 412 nm, designated as *A*_1_. For the control group, the Tris–glycine buffer (50 µL) was used in place of the Ellman’s reagent (50 µL), and the absorbance readings were taken as *A*_2_. Deionized water acted as a blank in the use of replacing the sample. The calculation was performed using Formula (2):

(2) $$R-\mathrm{SH}\;\mathrm{content}\;(\mu \mathrm{mol}/g)=\frac{73.53\times (A_{1}-A_{2})\times D}{C},$$

where *C*, *D*, and 73.53 correspond to the SPI concentration (mg/mL), dilution factor (in this experiment, which is 5), and the absorbance coefficient, respectively.

### 2.5. UV Spectra Scanning

The sample was diluted with deionized water to a concentration of 0.4 mg/mL. Spectral scanning was conducted using a UV-2600 spectrophotometer (UV-2600, SHIMADZU, Suzhou, China), covering the wavelength range of 200 to 400 nm, with a quartz cuvette of 10 mm diameter.

### 2.6. Endogenous Fluorescence Spectroscopy

The fluorescence spectra of the samples were measured using a fluorescence spectrophotometer (G9800A, Agilent Technologies, Inc., Santa Clara, CA, USA). With a sample concentration of 5 mg/mL, the excitation wavelength was maintained at 290 nm, while the emission was recorded from 280 to 500 nm. Both the excitation and emission slit widths were set at 5 nm.

### 2.7. FT-IR Spectroscopy

The infrared spectra of SPI and the SPI–polyphenol conjugates were recorded separately using a Fourier transform infrared spectrometer (FT-IR, Bruker, Ettlingen, Germany) at room temperature. All of the samples were freeze-dried, mixed with KBr, and pressed into thin slices before being recorded in the range of 500 to 4000 cm^−1^, utilizing 64 scans at a resolution of 4 cm^−1^.

### 2.8. Determination of Secondary Structure

The sample (0.05 mg/mL) was placed in a quartz cuvette with a path length of 1 mm, and the spectrum was recorded using a circular dichroism (CD) spectrometer (Chirascan, Glasgow, UK), scanning the range from 190 to 260 nm. An analysis of the sample’s four secondary structure proportions was conducted with the help of CDNN software (version 2.1).

### 2.9. Determination of Surface Hydrophobicity

The surface hydrophobicity (H_0_) of SPI and the SPI–polyphenol conjugates was determined using 8-aminoaniline-1-sulfonate (ANS) as a fluorescent probe, following the method described by Liu et al. [18]. The sample (4 mL) was diluted with deionized water to concentrations of 0.1, 0.3, 0.5, 0.7, and 0.9 mg/mL. The diluted samples were reacted with an ANS solution (20 μL, 8 mmol/L), and the fluorescence intensity was measured. The fluorescence settings were as follows: an excitation wavelength of 370 nm, an emission wavelength of 470 nm, and a slit width of 5 nm. The initial slopes of the fitting curves for the different protein concentrations and fluorescence intensities of each sample represent the surface hydrophobicity of the respective samples.

### 2.10. Determination of Emulsifying Properties

The emulsion activity index (EAI) and emulsification stability index (ESI) of the conjugates were calculated using a method described previously [19]. The sample (20 mL) was combined with soybean oil (5 mL) at a water-to-oil ratio of 4:1 (*v*:*v*) and homogenized at a speed of 10,000 r/min for 2 min to form an emulsion. Immediately after homogenization (0 min) and 10 min later, 50 μL was taken from the bottom of the emulsion and mixed with 8 mL of 0.1% SDS solution (*m*/*v*). The absorbance was measured at 500 nm using a UV-2600 spectrophotometer, with the SDS solution as a blank control. The following Formulae (3) and (4) were used to determine the EAI and ESI values:

(3) $$\mathrm{EAI}\;(m^{2}/g)=\frac{2.303*2*A_{0}*DF}{10000*\theta *C}$$

(4) $$\mathrm{ESI}\;(\min)=\frac{A_{0}*10}{A_{0}-A_{10}}$$

where *A*_0_ and *A*_10_ represent the absorbance of the emulsion at 0 min and 10 min, respectively; *DF* indicates the dilution factor of the sample with 0.1% SDS (which is 160 in this experiment); θ indicates the proportion of the sample to the soybean oil; C refers to the protein concentration, which is 10 mg/mL; and 2.303 represents the base of the natural logarithm.

### 2.11. Determination of Antioxidant Capacity

#### 2.11.1. DPPH Radical Scavenging Capacity

Following the method of Dai et al. [20], briefly, the sample (1 mL, 8 mg/mL) was combined with a DPPH–ethanol solution (3 mL, 1 mmol/L, with an absorbance less than 0.70) and allowed to react in the dark for 1 h. The absorbance of the sample was measured at 517 nm. The DPPH radical scavenging rate was calculated using Formula (5):

(5) $$\mathrm{DPPH}\;\mathrm{radical}\;\mathrm{scavenging}\;\mathrm{rate}\;(\%)=\{1-\frac{A_{1}-A_{2}}{A_{0}}\}\times 100$$

where *A*_0_, *A*_1_, and *A*_2_ represent the absorbance of the blank, the sample reacted with the DPPH–ethanol solution, and the sample reacted with the ethanol solution, respectively.

#### 2.11.2. ABTS Radical Scavenging Capacity

Following the method of Jiang et al. [21], the sample (0.64 mL, 0.625 mg/mL) was combined with an ABTS solution (4 mL, with a stock concentration of 7 mmol/L ABTS and 2.45 mmol/L K_2_S_2_O_8_, dissolved in a pH 7.4 phosphate buffer solution and diluted to an absorbance of 0.700 ± 0.02). The mixture was allowed to react at room temperature for 20 min, and the absorbance of the sample was measured at 734 nm. The ABTS radical scavenging capacity was calculated using Formula (6):

(6) $$\mathrm{ABTS}\;\mathrm{radical}\;\mathrm{scavenging}\;\mathrm{rate}\;(\%)=\{1-\frac{A_{1}-A_{2}}{A_{0}}\}\times 100$$

where *A*_0_, *A*_1_, and *A*_2_ represent the absorbance of the blank, the sample reacted with the ABTS solution, and the sample reacted with the PBS, respectively.

#### 2.11.3. Iron Ion Reducing Capacity

Following the method of Tan et al. [22], the sample (1 mL, 10 mg/mL) was combined with a phosphate buffer solution (3 mL, 0.2 mol/L, pH 6.6) and a K_3_[Fe(CN)_6_] solution (3 mL, 1% *w*/*w*), and heated in a water bath at 50 °C for 30 min. After cooling to room temperature, a trichloroacetic acid solution (3 mL, 10% *w*/*w*) was added, mixed, and allowed to stand for 30 min before centrifugation (5000 rpm, 10 min). The supernatant (2 mL) was then mixed with deionized water (3.5 mL) and a FeCl_3_ solution (0.5 mL, 0.1% *w*/*w*) and allowed to react at room temperature for 10 min. The absorbance was measured at 700 nm.

### 2.12. Statistical Analysis

The experiments in this study were repeated more than three times, and the data in the tables and figures are presented as the means ± standard deviations. The one-way analysis (ANOVA) and Duncan’s test for calculating the standard deviations and analyzing the significant differences were performed using SPSS (version 21.0.0.0, SPSS Inc., Chicago, IL, USA). All figures were prepared using OriginPro 2021 (version 9.8.0.200, OriginLab Co., Northampton, MA, USA).

## 3. Results and Discussion

### 3.1. Interaction of SPI–Polyphenol Conjugates

#### 3.1.1. Impact on Binding Capacity

**Figure 3:**
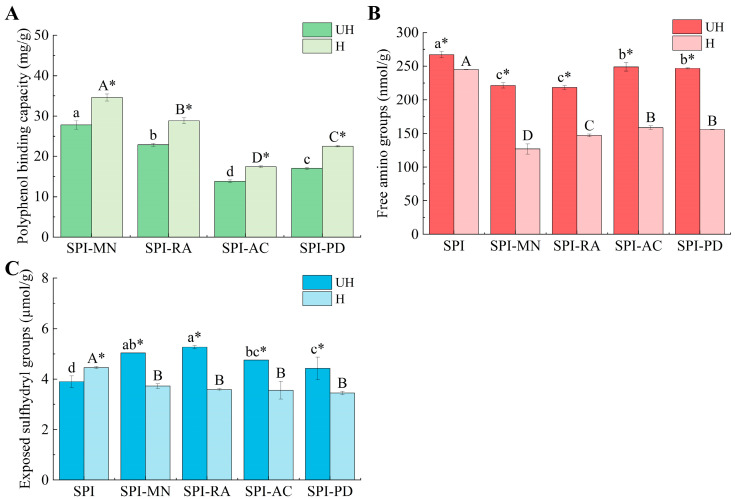
The polyphenol binding capacity (**A**), free amino groups (**B**), and exposed sulfhydryl groups (**C**) of SPI and the SPI–polyphenol conjugates with or without hydrodynamic cavitation. SPI means soy protein isolate; UH means the samples without hydrodynamic cavitation; H means the samples with hydrodynamic cavitation. There were significant differences (*p* < 0.05) among different uppercase letters with hydrodynamic cavitation, and the same uppercase letter means there was no significant difference (*p* > 0.05). There were significant differences (*p* < 0.05) among different lowercase letters without hydrodynamic cavitation, and the same lowercase letter means there was no significant difference (*p* > 0.05). * represents a significant difference between the protein–polyphenol conjugate groups of the same polyphenol before and after the cavitation treatment.

The binding capacity of polyphenols to proteins can clearly reflect the strength of the interactions between different polyphenols and SPI. As illustrated in Figure 3A, significant differences were observed in the binding amounts of the four polyphenols to the protein (*p* < 0.05), which might stem from the variability in the structure and morphology of the polyphenols, leading to differing degrees of interaction with the protein. Specifically, MN and RA exhibited higher binding amounts with SPI, while PD and AC demonstrated lower binding capacities. Notably, AC had the lowest binding amount with SPI, which might be attributed to its minimal phenolic hydroxyl content, leading to a lower affinity for the protein compared to the other three polyphenols. Wang et al. [15] also reported similar findings. When combined with perilla seed meal protein, gallic acid, protocatechuic acid, caffeic acid, apigenin, and luteolin showed varying degrees of binding, with protocatechuic acid, possessing the least phenolic hydroxyl groups, demonstrating the lowest level of binding to the proteins. They suggested that the lower phenolic hydroxyl content of PCA resulted in its lower reactivity when reacting with PSMP, leading to a significantly lower binding capacity of PCA compared to the other polyphenols.

After treatment with hydrodynamic cavitation, the binding capacity of polyphenols in the SPI–polyphenol conjugates was significantly higher than that before treatment (*p* < 0.05). This increase in binding capacity was attributed to the shear effects induced by hydrodynamic cavitation, which could disrupt hydrophobic interactions, leading to the unfolding of the protein structure and thereby exposing more active groups and binding sites that facilitate the association of polyphenols with proteins, ultimately enhancing the binding of polyphenols to SPI [23]. Furthermore, the hydroxyl radicals produced in the process of hydrodynamic cavitation were pivotal. On one hand, these radicals could oxidize polyphenols to quinones, which could then react with the amino or sulfhydryl groups present in the side chains of amino acids in the proteins, forming C-N or C-S bonds that promote the covalent binding of polyphenols to proteins. On the other hand, these radicals could also oxidize the amino acid residues in the protein side chains, subsequently generating protein radicals, which could react with polyphenols through C-N or C-S bonds to form polyphenol–protein covalent conjugates [24]. Xue et al. [5] found that the binding capacity of cyanidin-3-galactoside with SPI significantly increased after ultrasonic treatment, as such treatment produced hydroxyl radicals that attacked the hydrogen atoms of the hydroxyl and amino groups on the proteins, resulting in the formation of protein radicals that could covalently bind with polyphenols, leading to the formation of polyphenol–protein conjugates.

#### 3.1.2. Analysis of Free Amino and Exposed Sulfhydryl Content

The binding capacity of polyphenols to proteins can be further evaluated by measuring the levels of free amino groups and exposed sulfhydryl groups. As shown in Figure 3B, the free amino group content of the SPI–polyphenol conjugates was significantly lower than that of UH-SPI (*p* < 0.05). This reduction might result from the reaction between the polyphenols and the SPI amino groups, causing a diminution in the amino groups within the system. Additionally, the presence of SDS in the OPA reagent can disrupt the non-covalent interactions in the system; therefore, it was reasonable to deduce that covalent binding existed between the polyphenols and SPI in this experiment. The changes in the exposed sulfhydryl group content showed a different trend compared to those of the free amino groups. In comparison to UH-SPI, the SPI–polyphenol conjugates exhibited a significant increase (*p* < 0.05) in the content of exposed sulfhydryl groups. This elevation could be due to the structural changes in the protein resulting from its interaction with the polyphenols, causing SPI to unfold and reveal the previously concealed sulfhydryl groups within its structure. This was similar to the findings of Guo et al. [25], who discovered that the binding of rutin to whey protein isolate unfolded the protein molecular structure and increased the content of exposed sulfhydryl groups.

After the hydrodynamic cavitation treatment, the free amino group content of the SPI–polyphenol conjugates significantly decreased (*p* < 0.05), which might be due to the hydroxyl radicals generated during hydrodynamic cavitation promoting covalent binding between the polyphenols and SPI. Similar results were reported by Zhao et al. in their investigation on the interaction between whey protein and chlorogenic acid [26]. During the free radical grafting process, the oxidation reactions of hydrogen peroxide and ascorbic acid produced hydroxyl radicals, which facilitated the formation of C-N bonds between the amino groups and tryptophan residues of whey protein and the aromatic rings of chlorogenic acid, leading to a reduction in the amino group content within the system. After the hydrodynamic cavitation treatment, there was an increase in the content of exposed sulfhydryl groups in SPI, likely due to the structural unfolding induced by the process, thereby revealing more sulfhydryl groups. Nevertheless, the variations in the exposed sulfhydryl group content of the SPI–polyphenol conjugates followed a similar trend as those observed in the changes in the free amino group content. Compared to the untreated SPI–polyphenol conjugates, the treated conjugates showed a significant decrease in the content of exposed sulfhydryl groups (*p* < 0.05). This reduction might be attributed to the hydroxyl radicals generated by hydrodynamic cavitation promoting the formation of C-S bonds between the polyphenols and SPI. Similarly, Liu et al. [16] found that when ultrasound cavitation induced the binding of β-lactoglobulin with chlorogenic acid, the hydroxyl radicals generated by ultrasound cavitation could promote the formation of C-S bonds between β-lactoglobulin and chlorogenic acid, leading to a decrease in the sulfhydryl content.

### 3.2. Structural Properties of SPI–Polyphenol Conjugates

#### 3.2.1. UV Absorption Spectral Analysis

**Figure 4:**
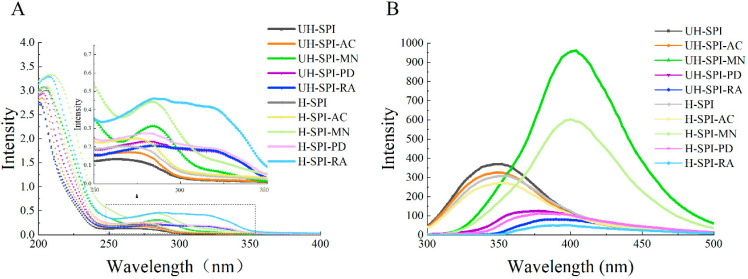
UV spectra (**A**) and endogenous fluorescence spectra (**B**) of SPI and SPI–polyphenol conjugates with or without hydrodynamic cavitation.

UV spectroscopy is a commonly used method for analyzing structural changes in proteins. The absorption of light by tryptophan and tyrosine residues results in an absorption peak for SPI at 280 nm [27]. As shown in Figure 4A, adding the polyphenols increased the intensity of the SPI absorption peak, which might be due to the benzene rings in the polyphenols that enhance UV light absorption [27]. This phenomenon further supported the idea of covalent and non-covalent interactions between the polyphenols and SPI. Dai et al. [28] found that the addition of catechin increased the absorption peak intensity, potentially due to an absorption band resulting from the π-π* transition of the indole ring on the tryptophan residue. Furthermore, the absorption peaks of the four SPI–polyphenol conjugates exhibited varying degrees of redshift, indicating a change in the microenvironment of the amino acid residues from non-polar to polar, which suggested that the polyphenols alter the structure of SPI [29]. Additionally, UH-SPI-RA and UH-SPI-PD displayed a distinct absorption peak at 320 nm, which differed from the UV spectrum of UH-SPI, indicating that structural changes occurred in SPI during the binding process. This finding was consistent with early research, which showed that a new absorption peak was also observed in the UV spectrum of the conjugate formed by the interaction between apigenin and perilla seed meal protein [15].

After undergoing the cavitation treatment, the SPI–polyphenol conjugates exhibited a notable increase in ultraviolet absorption peak intensity compared to their untreated state, which was attributed to the enhanced binding of the polyphenols and SPI facilitated by hydrodynamic cavitation. This led to an increase in the content of polyphenols and their benzene rings in the conjugate, resulting in a higher UV absorption peak intensity. Yu et al. [30] investigated the conjugate formed between hemp seed protein and chlorogenic acid and discovered that ultrasound treatment led to an enhancement in the absorption peak intensity of the conjugate. This increase might be due to the turbulence and shear forces generated by ultrasound, which disrupted the intermolecular interactions in the protein, exposing more amino acid residues.

#### 3.2.2. Endogenous Fluorescence Spectroscopic Analysis

Fluorescence spectroscopy is a technique used to analyze the molecular microenvironment, focusing primarily on studying the intrinsic tryptophan fluorescence in proteins. The intensity of intrinsic fluorescence can be used to assess the interaction between polyphenols and proteins [31]. The tryptophan residues serve as the main chromophores in SPI, and the aromatic ring structure can absorb light at specific wavelengths, leading to fluorescence [15]. As shown in Figure 4B, the fluorescence intensity undergoes obvious changes after the binding of the polyphenols with SPI without hydrodynamic cavitation treatment. Compared to UH-SPI, the fluorescence intensity of UH-SPI-RA, UH-SPI-AC, and UH-SPI-PD decreased, which might be due to the interaction between the tryptophan residues in SPI and the polyphenols, resulting in fluorescence quenching. This finding was consistent with the results of Yan et al. [32], which reported a noticeable reduction in fluorescence intensity after the covalent binding of SPI with epigallocatechin gallate. The interaction between the benzene rings of the polyphenols and the tryptophan residues might interfere with the normal electronic transition of the fluorescence chromophores, leading to fluorescence quenching. In contrast, the fluorescence intensity of UH-SPI-MN increased markedly compared to UH-SPI, possibly due to the exposure of more tryptophan and other chromophores within the protein after the binding of MN. Additionally, the maximum absorption peaks of the three polyphenols (MN, RA, and PD) exhibited a redshift, which was mainly attributed to the unfolding of the protein structure caused by the binding of the polyphenols to SPI, exposing the tryptophan residues to a more hydrophilic environment [33]. However, the maximum absorption peak of UH-SPI-AC did not exhibit a redshift, which might be due to the lower amount of AC bound to SPI, resulting in a lesser impact on the protein structure. This indicated that the structural differences among the polyphenols led to varying degrees of unfolding of the protein upon binding. Thus, the extent of the redshift in the maximum absorption peaks of the polyphenol–protein conjugates differed [34].

After the hydrodynamic cavitation treatment, the fluorescence intensity of the SPI–polyphenol conjugates decreased compared to the untreated group. This was primarily because hydrodynamic cavitation promoted the binding of polyphenols to proteins, making the fluorescence quenching phenomenon more pronounced. In comparison between the H group and the UH group, the maximum absorption peaks of H-SPI, H-SPI-AC, and H-SPI-PD showed a slight redshift, indicating that the abundant hydrophilic hydroxyl groups produced by hydrodynamic cavitation could facilitate the unfolding of the protein molecular structure, resulting in the tryptophan residues being transferred to a more hydrophilic environment. Research by Guo et al. [25] found that the effect of ultrasound treatment on rutin and whey protein isolate was similar, with the maximum absorption peak of the whey protein isolate–rutin conjugate shifting to 335 nm. They suggested that the ultrasound treatment affected the secondary structures of the proteins, leading to unfolding and an increase in the polarity of the environment surrounding the tryptophan. In contrast, the maximum absorption peaks of H-SPI-RA and H-SPI-MN exhibited a slight blueshift, indicating that under the influence of hydrodynamic cavitation, the amino acid residues of H-SPI-RA and H-SPI-MN were transferred to a more hydrophobic environment. Pan et al. [35] found that after the binding of chlorogenic acid, quercetin, rutin, and epigallocatechin gallate with bovine serum albumin, the microenvironment of the chromophores became more hydrophobic, resulting in varying degrees of blueshift in the maximum absorption peaks of the different protein–polyphenol conjugates compared to the control group. The occurrence of both redshifts and blueshifts in some complexes under hydrodynamic cavitation might stem from the different structures of the polyphenols, indicating that hydrodynamic cavitation had varying effects on the different polyphenols, leading to varied alterations in the microenvironment surrounding the chromophores within the protein–polyphenol conjugates.

#### 3.2.3. FT-IR Spectral Analysis

**Figure 5:**
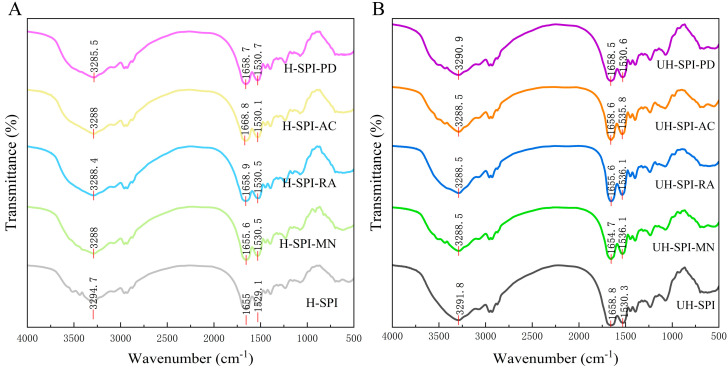
FT-IR spectra of H group (**A**) and UH group (**B**) of SPI and SPI–polyphenol conjugates.

In the FTIR spectrum, the stretching and bending vibrations of the protein peptide chain in the amide I region (1600–1700 cm^−1^) and amide II region (1500–1600 cm^−1^) may indicate different changes in the structure of proteins [36]. As shown in Figure 5, UH-SPI exhibited a strong absorption peak at 1658.8 cm^−1^, corresponding to the C=O stretching vibration in the amide I region. The peak of intense absorption at 1530.3 cm^−1^ arose from the stretching of the C-N bonds and the bending vibrations of N-H in the amide II region. The strong absorption peak at 3291.8 cm^−1^ was caused by the stretching vibrations of the hydroxyl or N-H groups [25]. After adding the polyphenols, the absorption peak of SPI at 3291.8 cm^−1^ exhibited varying degrees of redshift, while the peak at 1530.3 cm^−1^ showed varying degrees of blueshift. This might be attributed to the interactions between the polyphenols and the C-N, N-H, and COO- groups of SPI through hydrogen bonding, resulting in shifts in some of the absorption peaks of the SPI–polyphenol conjugate [37].

**Figure 6:**
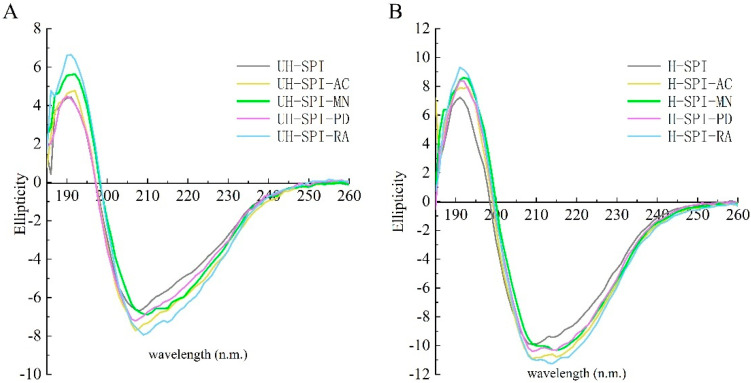
CD spectroscopy of UH group (**A**) and H group (**B**) of SPI and SPI–polyphenol conjugates.

After the hydrodynamic cavitation treatment, there were no clear changes in the absorption peak of the amide II region of SPI. The absorption peak at 3291.8 cm^−1^ underwent a blueshift to 3294.7 cm^−1^, while the absorption peak in the amide I region showed a redshift. This was mainly attributed to the C=O stretching vibrations and N-H bending vibrations, potentially resulting from the establishment of hydrogen bonds within the SPI molecules after the hydrodynamic cavitation treatment [38]. Compared to the SPI–polyphenol conjugates in the UH group, the SPI–polyphenol conjugates in the H group exhibited a significant redshift in the amide II region absorption peak, indicating that hydrodynamic cavitation promoted hydrophobic interactions between SPI and the polyphenols and altered the secondary structure of SPI (Figure 6) [39].

#### 3.2.4. Secondary Structure Analysis

CD spectroscopy enables the calculation and evaluation of protein secondary structures. Generally, intramolecular hydrogen bonds primarily stabilize the α-helix structure, and electrostatic interactions between amino acids can also influence it. The β-sheet structure is stabilized by hydrogen bonds between peptide chains, while the β-turn structure is formed by extended peptide chains creating 180° U-turns. Random coils represent unfolded conformations and are closely related to the flexibility of the protein [33]. Based on the results presented in Table 1, compared to UH-SPI, upon the binding of polyphenols to SPI, the structure of the SPI–polyphenol conjugates underwent significant changes (*p* < 0.05), reflected in a substantial increase in the content of α-helices. This increase might be due to the introduction of the polyphenols, which facilitated the formation of more stable hydrogen bonds between the amino acid side chains along the protein peptide chains, allowing the main chain of the peptide to twist into more right-handed helical structures. Meanwhile, the content of β-sheets significantly decreased compared to UH-SPI (*p* < 0.05), possibly because the interaction between the polyphenols and SPI made the internal environment of SPI more hydrophilic [24]. An unfolding of the protein structure was indicated by the decrease in β-turns and the corresponding increase in random coil structures. Zhang et al. [40] observed that after binding chlorogenic acid, vincristine, and ferulic acid with wheat germ albumin, the contents of α-helices and random coils in wheat germ albumin significantly increased, while the β-sheets and β-turns significantly decreased. They suggested that the polyphenols induced the protein structure to transition from ordered to disordered by increasing the content of α-helices and random coils, thereby significantly impacting the secondary structure of wheat germ albumin. Additionally, it could be observed from Table 1 that the changes in secondary structures among the different SPI–polyphenol conjugates were significantly different (*p* < 0.05), which might be attributed to the variation in the polyphenol structures and the content of phenolic hydroxyl groups [41].

After the hydrodynamic cavitation treatment, the changes in the secondary structures of the SPI–polyphenol conjugates became more pronounced, evidenced by a significant increase in the content of α-helices, β-turns, and random coils in the SPI–polyphenol conjugates compared to H-SPI, with an even more significant increase than that seen in the UH group of SPI–polyphenol conjugates (*p* < 0.05). The content of β-sheets also decreased significantly (*p* < 0.05). This was due to the cavitation effects caused by hydrodynamic cavitation, which led to a reduction in the degree of protein aggregation [42], thus facilitating the formation of hydrogen bonds between the polyphenols and proteins. Dai et al. [38] also reported similar findings, noting that after ultrasound treatment, the relative content of α-helices and β-sheets in SPI–catechin decreased more significantly, and the relative content of β-turns and random coils increased more markedly. This was because the ultrasound treatment relaxed the structure of the conjugate, exposing the internal groups of the protein peptide chains to the surface and enhancing the interactions between SPI and catechin.

#### 3.2.5. Evaluation of H_0_

**Figure 7:**
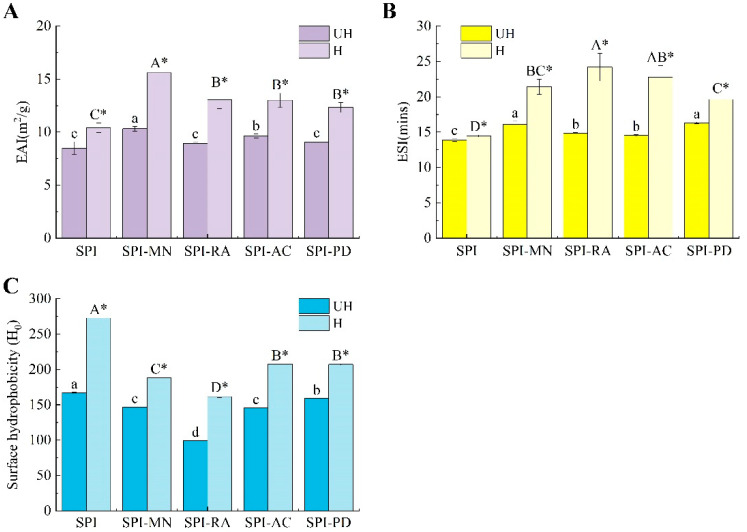
The EAI (**A**), ESI (**B**), and H_0_ (**C**) of SPI and the SPI–polyphenol conjugates with or without hydrodynamic cavitation. SPI means soy protein isolate; UH means the samples without hydrodynamic cavitation; H means the samples with hydrodynamic cavitation.There were significant differences (*p* < 0.05) among different uppercase letters with hydrodynamic cavitation, and the same uppercase letter means there was no significant difference (*p* > 0.05). There were significant differences (*p* < 0.05) among different lowercase letters without hydrodynamic cavitation, and the same lowercase letter means there was no significant difference (*p* > 0.05). * represents a significant difference between the protein–polyphenol conjugate groups of the same polyphenol before and after the cavitation treatment.

The H_0_ of a protein is positively correlated with the number of hydrophobic groups exposed on the protein surface, reflecting changes in the protein’s tertiary structure and closely related to the protein’s solubility, emulsifying properties, and other characteristics. As shown in Figure 7C, the addition of the polyphenols led to a significant decrease in the H_0_ of SPI (*p* < 0.05). There were two possible reasons for this phenomenon: first, the reduction in the number of hydrophobic sites after SPI bound with the polyphenols lowered the extent of the ANS binding and consequently resulted in a decrease in H_0_. Second, the hydroxyl groups present in the polyphenol structure might render the environment around SPI more hydrophilic, and a hydrophilic environment could affect the electronic excitation of the ANS probe, leading to a lower H_0_ of the SPI–polyphenol conjugate [43]. Similarly, the study by Wen et al. [42] found that the addition of chlorogenic acid altered the aggregation morphology of ovalbumin, with the H_0_ of ovalbumin gradually decreasing as the concentration of chlorogenic acid increased. Among all samples, the H_0_ of UH-SPI-RA decreased most significantly (*p* < 0.05), possibly because RA had the highest number of active phenolic hydroxyl groups, leading to the most hydrophilic environment around SPI. In contrast, UH-SPI-PD showed the slightest decrease in H_0_, likely because PD has a more complex structure than the other three polyphenols, resulting in greater steric hindrance. Its addition not only introduced phenolic hydroxyl groups, but also unfolded the structure further, exposing hydrophobic groups; thus, the decrease in H_0_ is relatively less.

After the hydrodynamic cavitation treatment, the H_0_ of SPI significantly increased (*p* < 0.05), likely because the cavitation effect loosened the SPI structure, exposing more hydrophobic groups. This finding was consistent with the report by Ji et al. that the H_0_ of pea protein increased after high-pressure microjet treatment [44]. Compared to H-SPI, the H_0_ of the SPI–polyphenol conjugates decreased significantly after hydrodynamic cavitation (*p* < 0.05), which might be attributed to the hydroxyl free radicals generated by hydrodynamic cavitation promoting the binding of polyphenols to SPI. The hydrophilic groups of the polyphenols contributed to the increased hydrophilicity of the SPI–polyphenol conjugate and reduced the H_0_ [45]. However, the H_0_ of the SPI–polyphenol conjugates after cavitation was significantly higher than that of the untreated conjugates (*p* < 0.05), likely because hydrodynamic cavitation facilitated the interactions between the polyphenols and SPI, leading to a more unfolded structure and further exposing the hydrophobic groups.

### 3.3. Functional Properties of SPI–Polyphenol Conjugates

#### 3.3.1. Evaluation of Emulsifying Properties

The emulsifying property of proteins is one of their essential characteristics. As emulsifiers, proteins interact with oil phases through their hydrophobic groups, while their hydrophilic groups interact with water phases, thereby forming emulsions at the oil–water interface [46]. The EAI and ESI signify the capacity of proteins to create and maintain emulsions at the oil–water boundary, respectively. Figure 7A illustrated that both UH-SPI-MN and UH-SPI-AC exhibited a significant improvement in EAI compared to UH-SPI (*p* < 0.05), while the EAI of UH-SPI-RA and UH-SPI-PD showed a slight increase. This improvement might be attributed to the increased random coil structures during the binding process between the polyphenols and proteins, indicating an enhanced protein flexibility that facilitates emulsion formation, thereby improving the emulsifying properties [47]. At the same time, the ESI (Figure 7B) of all four SPI–polyphenol conjugates was also significantly enhanced compared to UH-SPI (*p* < 0.05). This enhancement might be due to the incorporation of the polyphenols, which made the SPI structure more relaxed, promoting an organized rearrangement at the oil–water interface and giving the interface a certain elasticity, which reduced the surface tension [48]. Yan et al. [49] also reported similar results that the EAI and ESI significantly increased after the combination of SPI with gallic acid. This improvement was attributed to the polyphenols enhancing the flexibility and surface hydrophobicity of the protein, thus improving the protein’s ability to reach equilibrium at the oil–water interface, resulting in a more stable interface.

After the hydrodynamic cavitation treatment, both the EAI and ESI of the SPI–polyphenol conjugates significantly increased (*p* < 0.05), which was due to the intense shear forces generated by hydrodynamic cavitation that led to a greater unfolding of the protein structure, exposing more hydrophobic groups. Some of these exposed hydrophobic groups were replaced by the polyphenols, while others enhanced the protein’s adsorption capacity at the oil–water interface, improving the emulsifying properties of the protein. This finding was consistent with Yu’s previous research on hempseed protein, where the author attributed the improvement in the emulsifying performance to ultrasound treatment, which promoted the binding of chlorogenic acid with the protein, increasing the exposure of aromatic residues [30]. This led to the enhanced affinity of the conjugate at the oil–water interface, resulting in an improved emulsifying performance.

#### 3.3.2. Evaluation of Antioxidant Capacity

**Figure 8:**
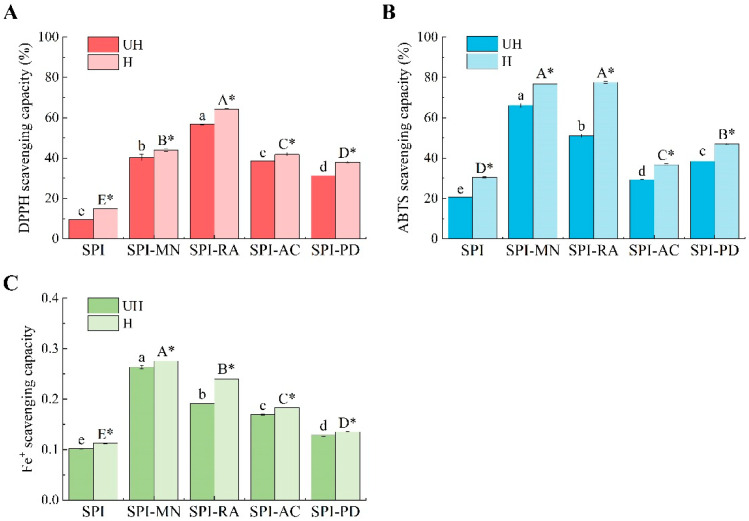
The DPPH radical scavenging capacity (**A**), ABTS radical scavenging capacity (**B**), and iron ion reduction capacity (**C**) of SPI and the SPI–polyphenol conjugates with or without hydrodynamic cavitation. SPI means soy protein isolate; UH means the samples without hydrodynamic cavitation; H means the samples with hydrodynamic cavitation. There were significant differences (*p* < 0.05) among different uppercase letters with hydrodynamic cavitation, and the same uppercase letter means there was no significant difference (*p* > 0.05). There were significant differences (*p* < 0.05) among different lowercase letters without hydrodynamic cavitation, and the same lowercase letter means there was no significant difference (*p* > 0.05). * represents a significant difference between the protein–polyphenol conjugate groups of the same polyphenol before and after the cavitation treatment.

The antioxidant performance of the samples can be evaluated by measuring the DPPH and ABTS scavenging capacities and the iron ion reduction capacity of the SPI–polyphenol conjugates. Generally, the numerous hydroxyl groups in polyphenolic structures exhibit significant antioxidant activity [50]. Figure 8 displays the results from the three methods; compared to UH-SPI, the DPPH and ABTS radical scavenging capacities, as well as the iron ion reduction capacity, of the SPI–polyphenol conjugates were significantly improved (*p* < 0.05). Wang et al. [15] also found similar results, showing that the antioxidant properties of protein–polyphenol conjugates were significantly higher than those of perilla seed meal proteins. This enhancement might be attributed to the excellent antioxidant properties of polyphenols. When the polyphenols were combined with proteins to produce conjugates, the conjugates could more effectively capture and scavenge free radicals, thus enhancing the antioxidant capacity. As illustrated in Figure 8A, UH-SPI-RA exhibited the highest DPPH radical scavenging capacity, likely because it contained the highest level of active phenolic hydroxyl groups among the RA molecules. UH-SPI-MN showed the highest ABTS radical scavenging capacity and iron ion reduction capacity, likely because of the highest binding quantity of MN and its smaller molecular weight, allowing it to remove ABTS radicals and reduce iron ions more effectively [47]. In contrast to the DPPH radical scavenging capacity and iron ion reduction capacity, the ABTS radical scavenging capacity of UH-SPI-AC was lower than that of UH-SPI-PD. This could be due to the largest molecular weight of AC, which led to higher steric hindrance, resulting in the reduced reactivity of the conjugate towards ABTS radicals [51].

Compared to the UH group, the DPPH and ABTS radical scavenging capacities and the iron ion reduction capacity of all SPI–polyphenol conjugates after the hydrodynamic cavitation treatment were significantly increased (*p* < 0.05). This enhancement was attributed to the hydroxyl radicals generated by the cavitation effect, which promoted the interaction between the polyphenols and SPI. The conjugates treated by hydrodynamic cavitation exhibited a higher binding capacity of polyphenols, thereby introducing additional phenolic hydroxyl groups and ultimately enhancing their antioxidant capabilities [52]. However, Iscimen et al. [36] reported different results, finding that the DPPH and ABTS radical scavenging capacities of faba bean protein–grape leaf polyphenol conjugates decreased after ultrasound treatment. This was attributed to the excessive free radicals generated by high-power ultrasound, leading to the oxidative degradation of the phenolic compounds and a reduction in the antioxidant properties of the conjugates. In addition, the DPPH radical scavenging capacity of the solvent is related to its polarity [53]. In this experiment, the samples were dissolved in an ethanol solution to measure their DPPH radical scavenging capacities. Therefore, the increased antioxidant capacity of the SPI–polyphenol conjugates after the cavitation treatment might be due to the enhanced solubility of the conjugates in the ethanol solution following hydrodynamic cavitation.

## 4. Conclusions

Two types of lignan polyphenols (MN, AC) and two types of stilbene polyphenols (RA, PD) have exhibited varying degrees of binding with SPI. Among them, MN and AC exhibited relatively higher binding amounts with SPI, and the resulting SPI-MN and SPI-AC conjugates demonstrated good antioxidant activity and emulsifying properties. Hydrodynamic cavitation treatment could cause the SPI structure to unfold, increasing the content of exposed sulfhydryl groups and the H_0_, thereby exposing more sites for interaction with the polyphenols. Additionally, the free radicals generated by hydrodynamic cavitation could promote covalent binding between the polyphenols and SPI, resulting in a significant increase in the binding amounts of the four polyphenols used in the experiment with SPI. The UV spectroscopy, fluorescence spectroscopy, and CD spectroscopy results indicated that the addition of these four polyphenols altered the structure of SPI, and the hydrodynamic cavitation treatment made these structural changes more pronounced. Furthermore, the SPI–polyphenol conjugates treated by hydrodynamic cavitation exhibited significantly enhanced antioxidant activity and emulsifying properties compared to those that were untreated. This demonstrated that the application of hydrodynamic cavitation technology offered a new approach for the preparation of protein–polyphenol conjugates. The prepared SPI–polyphenol conjugates can be used to construct emulsion delivery systems for the functional food industry.

## Figures and Tables

**Table 1:** Content of secondary structure content of SPI and SPI–polyphenol conjugates with or without hydrodynamic cavitation.

Material	α-Helix/%	β-Sheet/%	β-Turn/%	Random Coil/%
UH-SPI	18.49 ± 0.29 ^e^	34.54 ± 0.30 ^a*^	17.56 ± 0.05 ^a^	29.71 ± 0.13 ^c*^
UH-SPI-MN	20.41 ± 0.09 ^c^	30.52 ± 0.10 ^d*^	17.23 ± 0.03 ^c^	31.81 ± 0.08 ^a^
UH-SPI-RA	21.68 ± 0.22 ^a^	30.56 ± 0.12 ^d*^	17.32 ± 0.03 ^bc^	30.08 ± 0.23 ^b^
UH-SPI-AC	19.97 ± 0.14 ^d^	32.85 ± 0.12 ^b*^	17.52 ± 0.03 ^a^	29.72 ± 0.12 ^c^
UH-SPI-PD	21.23 ± 0.14 ^b^	31.00 ± 0.11 ^c*^	17.39 ± 0.10 ^b^	30.15 ± 0.05 ^b*^
H-SPI	24.92 ± 0.23 ^E*^	27.86 ± 0.31 ^A^	17.88 ± 0.04 ^C*^	29.02 ± 0.16 ^D^
H-SPI-MN	29.39 ± 0.56 ^C*^	20.13 ± 0.41 ^C^	18.16 ± 0.11 ^AB*^	33.32 ± 0.18 ^A*^
H-SPI-RA	33.29 ± 0.43 ^A*^	18.25 ± 0.32 ^D^	18.18 ± 0.06 ^AB*^	30.95 ± 0.17 ^B*^
H-SPI-AC	31.56 ± 0.29 ^B*^	19.93 ± 0.05 ^C^	18.24 ± 0.06 ^A*^	30.82 ± 0.21 ^B*^
H-SPI-PD	27.36 ± 0.18 ^D*^	24.72 ± 0.31 ^B^	18.10 ± 0.07 ^B*^	29.85 ± 0.22 ^C^

The data in the table are the means ± standard deviations. There were significant differences (*p* < 0.05) among different uppercase letters with hydrodynamic cavitation, and the same uppercase letter means there was no significant difference (*p* > 0.05). There were significant differences (*p* < 0.05) among different lowercase letters without hydrodynamic cavitation, and the same lowercase letter means there was no significant difference (*p* > 0.05). * represents a significant difference between the protein–polyphenol conjugate groups of the same polyphenol before and after the cavitation treatment.

## Data Availability

The original contributions presented in the study are included in the article, further inquiries can be directed to the corresponding author.
